# Neuropsychiatric symptoms of multiple sclerosis: A case report

**DOI:** 10.1192/j.eurpsy.2021.1264

**Published:** 2021-08-13

**Authors:** P. Espada Dos Santos, J. Facucho-Oliveira, D. Esteves-Sousa, M. Costa, M. Albuquerque, A. Fraga, M. Marinho, P. Cintra

**Affiliations:** Psiquiatria E Saúde Mental, Hospital de Cascais Dr. José de Almeida, Alcabideche, Portugal

**Keywords:** psychosis, Multiple sclerosis, Psychiatric disorders, Interferons

## Abstract

**Introduction:**

Multiple Sclerosis (MS) is an immune-mediated inflammatory demyelinating disease of the central nervous system. Concomitant psychiatric diseases are frequent in MS, with depression and anxiety disorders constituting the majority. The presence of psychotic disorders with MS is rare. Several studies have reported that psychotic symptoms usually develop after the neurological signs of MS and they are mostly linked to the side effects of treatment with interferon or with corticosteroids.

**Objectives:**

The authors report here the case of patient with MS without psychiatric history that developed psychotic symptoms.

**Methods:**

Beside the medical record of the patient a non-systematic search of the literature was carried out in the databases Pubmed and Google Scholar with the terms “Multiple Sclerosis”, “Multiple Sclerosis treatment ”and“ Neuropsychiatric symptoms ”.

**Results:**

A 38 years old woman with MS, with no psychiatry history developed paranoid and reference delusions, several months after starting interferon beta-1a therapy. The inferferon therapy was stopped and the patient was started Risperidone 3 mg id with a rapid but only partial remission of the psychotic symptoms. The patient presented high blood levels of prolactine and the MRI showed a pituitary microadenome. The Risperidone was switched to Aripiprazol 15 mg also with partial remission of the psychtic symptons.

**Conclusions:**

It is not possible to attribute our patient’s psychotic symptoms entirely to his Interferon therapy or to MS lesion load, but the occurrence during treatment, no psychiatric history and the rapid but parcial resolution with discontinuing suggest that Interferon therapy was at least contributory to the clinical picture.

